# Enzyme-inducing effect of the original anticonvulsant on the liver monooxygenase system – prospects for clinical use in the treatment of paroxysmal disorders

**DOI:** 10.1192/j.eurpsy.2025.2094

**Published:** 2025-08-26

**Authors:** E. I. Mochalova, T. V. Shushpanova, E. V. Gutkevich, T. P. Novozheeva, O. V. Shushpanova, I. N. Smirnova, N. E. Kolomiets

**Affiliations:** 1 Mental Health Research Institute, Tomsk National Research Medical Center, Russian Academy of Sciences; 2 National Research Tomsk State University, Tomsk; 3 Mental Health Research Center, Moscow; 4 Tomsk Research Institute of Balneology and Physiotherapy, branch of the Federal Scientific and Clinical Center for Medical Rehabilitation and Balneology of the Federal Medical and Biological Agency; 5 Siberian State Medical University of the Ministry of Health of the Russia, Tomsk; 6 Kemerovo State Medical University of the Ministry of Health of the Russia, Kemerovo, Russian Federation

## Abstract

**Introduction:**

It is necessary to know how the pharmacokinetics of psychotropic drugs changes in the body of patients during their long-term use.

**Objectives:**

To study the pharmacokinetic parameters of the anticonvulsant galodif in experimental rats at different times of drug administration.

**Methods:**

Galodif (meta-chlorobenzhydryl urea, 100 mg/kg) was administered in rats (in suspension, intragastrically) for: 1, 5 and 15 days. Galodif was determined in the microsomal fraction of rat liver. Pharmacokinetic parameters were calculated by a model-independent method of statistical moments. The statistical significance of differences was assessed using the Kolmogorov-Smirnov λ-test at p<0.05.

**Results:**

A single administration of galodif to rats is accompanied by a slowdown in its elimination from the body: the values of T1/2 (18.82±6.25h), MRT (22.41±7.07h), MET (10.64±2.84h), AUC (15.01±4.86 mcg/ml) increase, indicating the retention of galodif in the body tissues. A 5-fold administration of galodif stimulates the elimination of the drug: T1/2 (2.22±0.52*h), AUC (3.68±0.79*mcg/ml), MRT (2.95±0.73*h), MET (3.00±0.65*h) decrease. The pharmacokinetic parameters of the drug indicate a pronounced tissue availability of galodif molecules. With a 15-fold administration of galodif, the elimination of the drug from the body slows down somewhat, remaining accelerated relative to a single administration: T1/2 (10.79±2.90*h), MRT (3.97±1.03*h), MET (12.05±4.10*h), AUC (19.28±7.13*mcg/ml). The revealed changes in kinetic parameters during long-term administration of galodif to rats stimulate the elimination of the drug by inducing the microsomal liver oxidative system.

**Conclusions:**

The choice of a drug that combines anticonvulsant and detoxifying properties is of great importance in long-term therapy of paroxysmal disorders, epilepsy and alcoholism.

**Disclosure of Interest:**

None Declared

